# Clinical efficacy of antibiotic-loaded bone cement and negative pressure wound therapy in multidrug-resistant organisms diabetic foot ulcers: a retrospective analysis

**DOI:** 10.3389/fcimb.2024.1521199

**Published:** 2025-01-03

**Authors:** Huihui Guo, Zhenqiang Xue, Siwei Mei, Tengfei Li, Haiyang Yu, Tao Ning, Yongbin Fu

**Affiliations:** Department of Orthopedics, Fuyang City People’s Hospital, Fuyang, China

**Keywords:** multidrug-resistant organisms, diabetic foot ulcers, antibiotic-loaded bone cement, vacuum sealing drainage, clinical efficacy

## Abstract

**Objective:**

The purpose of this study was to investigate the clinical efficacy of antibiotic-loaded bone cement (ALBC) combined with Negative pressure wound therapy (NPWT) aspiration technique in the treatment of multidrug-resistant diabetic foot ulcers (MDRO-DFUs).

**Methods:**

A retrospective analysis of the clinical data of 80 patients with MDROs-DFU who were used Vacuum sealing drainage (VSD) as NPWT excipient and met the inclusion criteria from January 2019 to January 2024 at our hospital. Patients were divided into an experimental group and a control group, with 40 cases in each. The control group received conventional treatment, routine debridement, and NPWT treatment, while the experimental group received ALBC treatment in addition to the treatment plan of the control group. Measurements of blood inflammatory indicators, foot hemodynamic indicators, wound bacterial clearance time, wound healing time, and hospital stay were taken before and after treatment for both groups.

**Results:**

Inflammatory indexes, Vascular endothelial growth factor(VEGF), and internal diameter of dorsalis pedis arteriosus of both groups after treatment were significantly better than those before treatment, and the improvement of the experimental group was more obvious than that of the control group; the experimental group had a significantly shorter time of trauma bacterial turnover, healing time of trauma, and hospitalization time compared with that of the control group (P<0.05).

## 1 Background

The global prevalence of diabetes is currently estimated at around 537 million individuals, with projections indicating a potential increase to 783 million by the year 2045 (Ahmad et al., 2022). The diabetic foot is one of the most serious complications of diabetes mellitus, and about 1/3 of diabetic patients are at risk of infection (Deng et al., 2023; McDermott et al., 2023), leading to infections, ulcers, or tissue destruction around the ankle. This condition often occurs with peripheral neuropathy or varying degrees of arterial blockages in the lower limbs (van Netten et al., 2020). DFUs frequently manifest in elderly patients with a prolonged history of diabetes. These individuals commonly exhibit varying degrees of stenosis or occlusive lesions in peripheral blood vessels, often concomitant with neurological and vascular pathologies (Abdissa et al., 2020). Research indicates that neuropathy serves as a primary catalyst in ulcer formation. The impaired sensation in the foot, coupled with inadequate preventive measures against abnormal pressure, predisposes these patients to infections, thereby exacerbating the development of ulcers (Liu et al., 2022). Following the onset of ulcers, which frequently do not receive adequate initial attention, the affected the area and depth tend to expand, potentially extending to the bone. This progression is often accompanied by a polymicrobial infection, presenting significant challenges in clinical management. Following the initial formation of an ulcer, which typically receives minimal treatment, the lesion enlarges and deepens, potentially spreading to the bone. This development is frequently accompanied by a polymicrobial illness, which creates considerable therapeutic issues. According to reports, the majority of DFUs infections display multidrug resistance, especially among gram-positive organisms, with staphylococci being the prevalent pathogens (Coskun et al., 2024; Guo et al., 2023; Morton and Coghill, 2024; Wu et al., 2018). The increasing misuse of antibiotics has led to a rise in the number of patients suffering from the Multidrug-resistant organisms (MDROs) infections, complicating treatment efforts (Du et al., 2022; Yang et al., 2024). These patients frequently experience prolonged hospital stays and incur significant medical expenses. In severe instances, the level of necrosis and infection may become uncontrollable, necessitating amputation, which can pose life-threatening risks (Armstrong et al., 2023; Hung et al., 2024; Quilici et al., 2016).

MDROs are prevalent pathogens in patients with DFUs infections (Guo et al., 2023; Yang et al., 2024). This issue is especially concerning among DFUs patients infected with pathogenic organisms, as the increasing prevalence of MDROs is largely attributed to the misuse of antibiotics. However, managing MDROs-DFUs presents a significant challenge for clinicians due to severe ulcer ischemia, extensive tissue necrosis, and infection with MDROs. Conventional treatments are frequently insufficient, necessitating a multidisciplinary approach incorporating vascular surgery, endocrinology, infectious disease management, orthopedics, and other relevant fields (Armstrong et al., 2023; Bloomgarden, 2023). For the treatment of classic DFUs, most clinicians prefer surgical removal of diseased the tissue or bone as the primary strategy, which is supplemented by systemic antibiotic medication during the procedure (Ramachandran et al., 2023). The fundamental treatment principles for DFUs include surgical debridement, tibial bone transfer to facilitate wound healing (Kong et al., 2024; Qin et al., 2023; Yuan et al., 2021), infection control, enhancement of local vascular perfusion, promotion of wound healing, and prevention of amputation (Chang and Nguyen, 2021; Chen et al., 2024). However, there is no consensus on how to manage MDROS-DFUs (Senneville et al., 2024; Wang et al., 2024). Treatment for MDROS-DFUs not only includes wound debridement but also wound infection and MDRO management.

Negative pressure wound therapy(NPWT)is the commonly utilized for open wounds and soft tissue infections (Ji et al., 2021). The NPWT can enable fluid drainage via continuous negative pressure suction while providing a somewhat clean environment to improve wound healing and reduce infection risk. According to reports, NPWT is indicated for the treatment of DFUs due to its influence on wound drainage and repair (Apelqvist et al., 2017). However, NPWT alone does not have antibacterial properties and cannot kill bacteria (Wu et al., 2023), limiting its clinical applications.

Antibiotic-loaded bone cement (ALBC) serves as a stable carrier for antibiotics, maintain high concentrations of antibiotics in infected tissues for long periods of time. It has been widely utilized in clinical infection prevention and treatment (Hohendorff et al., 2019; Namba et al., 2020). Research indicates that ALBC not only reduces infection rates during joint replacement but plays a crucial role in treating soft tissue wounds (Mendame et al., 2021; Sebastian et al., 2020; Tarabichi and Parvizi, 2023). ALBC may hold sustained and effective clinical value in the treatment of DFU. Limited studies exist on the combination of ALBC with NPWT for treating MDROs-DFUs infections. This study aims to retrospectively analyze the clinical efficacy and experience of using ALBC combined with NPWT in our hospital for treating MDROs-DFUs infections.

## 2 Materials and methods

### 2.1 General information

This retrospective study enrolled 80 patients diagnosed with DFUs who received treatment from January 2019 to January 2024 and met the predefined inclusion criteria. In this study, VSD(Wuhan Visdi Medical Technology Co, Model and Specification: VSD-D-2-15*10*1.)was used as NPWT excipient. All participants provided informed consent in accordance with the stipulations established by the ethics committee of the authors’ affiliated institution. Employing the Meggitt-Wagner classification system, all patients were categorized as having Wagner grades 2 to 4 DFUs, with lower limb vascular lesions assessed by a single vascular surgeon.

The Diagnostic Criteria for Diabetic Foot Ulcers: The diagnostic criteria for diabetic foot ulcers are based on a thorough clinical examination that includes at least two signs of inflammation, such as erythema, elevated temperature, edema, and discomfort. Additionally, the possibility of suppuration, fluctuation, or lymphangitis should be evaluated (Cortes-Penfield et al., 2023; Wukich et al., 2024).

Multidrug-resistant organisms refer to bacteria that show resistance to three or more classes of antibiotics used in clinical practice (Kandemir et al., 2007).

### 2.2 Inclusion and exclusion criteria

Inclusion Criteria: (1) Patients with a verified diagnosis of type 2 diabetes mellitus who present with diabetic foot ulcers defined as Wagner grade 2 or above. (2)Patients with chronic infected wounds that have persisted for more than two weeks and are expected to require negative pressure wound therapy; (3)Patients who agree to refrain from alternative treatments during the active phase of the study and have comprehensive clinical data; and (4)Patients with MDROs as determined by drug sensitivity testing.

Exclusion criteria include: (1) non-diabetic foot infections, such as pressure ulcers, vasculitis, gangrenous pyoderma, and other chronic infections; (2) suspected or confirmed allergy to bone cement components; (3) patients with abnormal coagulation profiles; (4) patients with acute deep vein thrombosis; (5) concurrent malignant tumors; (6) patients with sepsis; (7) coexisting hematological disorders other than anemia; and (8) patients with incomplete clinical data.

### 2.3 Observation indicators

The clinical data of the patients were gathered, including their age, gender, ankle-brachial index (ABI), diabetes duration, HbA1c, Wagner classification, hospitalization period, and wound healing time. Bacterial culture strains are classified as Gram-positive (G+), Gram-negative (G-), and mixed bacteria. The number of positive wound bacterial cultures in the two groups before treatment, as well as the second, fourth, eighth, and sixteenth days following therapy, was counted. Color Doppler ultrasound was used before and after therapy to measure vascular diameter in the dorsal foot, serum vascular endothelial growth factor (VEGF) content, and levels of interleukin-6 (IL-6) and ESR CRP. The DFUs patient database has been entered and set up. The differences in each index between the two groups were investigated and analyzed to determine their statistical significance.

### 2.4 Data analysis

SPSS 25.0 statistical software was used to process the data. Intergroup data comparisons were conducted using t-tests for normally distributed variables, Mann-Whitney U tests for non-parametric variables, and either chi-square tests or Fisher’s exact test as appropriate. The difference was considered statistically significant at P < 0.05.

## 3 Treatment

### 3.1 Preoperative treatment

Upon admission, comprehensive internal medicine treatment is initiated, with routine consultations from endocrinology specialists to manage blood glucose levels, aiming to maintain fasting blood glucose below 7 mmol/L. Secretions from ulcerative wounds are subjected to bacterial culture and antimicrobial susceptibility testing to guide antibiotic therapy. Preoperatively, symptomatic supportive treatment is provided to improve nutritional status. Nursing care includes enhanced patient education, advising against excessive limb activity, and recommending smoking and alcohol cessation.

### 3.2 Surgical treatment

All patients had their surgeries performed by the same chief surgeon. Before debridement, the ulcer wound was cleaned and the bacterial culture of the ulcer discharge or pus was taken. The surgery was founded on the concepts of wound exposure, thorough debridement, pus removal, and smooth drainage. There is no universal incision standard for surgery, depending on the amount of infection discovered during intraoperative exploration. The infected necrotic soft tissue was removed, and the wound was periodically washed with hydrogen peroxide, iodine, and saline before being debrided until fresh leaking tissue developed; when bone infection was present, the bone resection was moderately increased. The VSD dressing was appropriately trimmed to match the size of the wound, then applied over the foot ulcer and secured with intermittent sutures. The skin surrounding the ulcer was cleaned, followed by the application of a permeable film using an imbrication technique. After achieving satisfactory sealing, the drainage tube was secured using a mesangial method. Negative pressure should be maintained at 80-125 mmHg (1 mmHg = 0.133 kPa), ensuring that the filled dressing exhibits significant collapse, allowing visibility of the drainage tube shape without any fluid accumulation beneath the film. Postoperative irrigation of ulcers is recommended for a duration of 7 to 10 days. On this basis, patients in the experimental group were treated with antibiotic bone cement in combination, and 2 g of vancomycin (VACOCIN 0.5 g/branch) was added into 40 g of bone cement before operation, mixed well and made into dough It was mixed well and made into a dough-like shape for standby. In the experimental group, after removing the inflammatory granulation tissue of the foot trauma, the appropriate amount of antibiotic bone cement was inserted into the trauma according to the size of the foot trauma. In order to avoid damage to the soft tissues caused by the heat generated by the bone cement, the bone cement is left in the air before it generates heat, and is then molded into the shape of the wound defect and inserted into the wound after the heat is released. The area covered by the bone cement is slightly larger than the area of the soft tissue defect. Before hardening of the bone cement, a 2.0-mm needle was used to poke an appropriate number of holes in the bone cement for adequate drainage. When the bone cement was hot, it was washed with saline to cool down the temperature, and then the bone cement was fixed on the wound with silk suture. Then VSD dressing was performed to cover it and form a closed environment.

### 3.3 Postoperative management

Postoperatively, it is imperative to maintain strict control over the patient’s blood glucose levels and continuously monitor their overall clinical status. Symptomatic treatment should be administered as necessary, alongside counseling for smoking cessation and alcohol abstinence. Health education must be provided to the patient, with adjustments made to intravenous administration of sensitive antibiotics once bacterial culture results are obtained. Additionally, secretions will be collected for bacterial culture analysis on postoperative days 3, 7, and 14, following a cycle of every 7 days until cultures yield negative results.

## 4 Results

### 4.1 Comparison of general condition between two groups of patients

**Figure 1:**
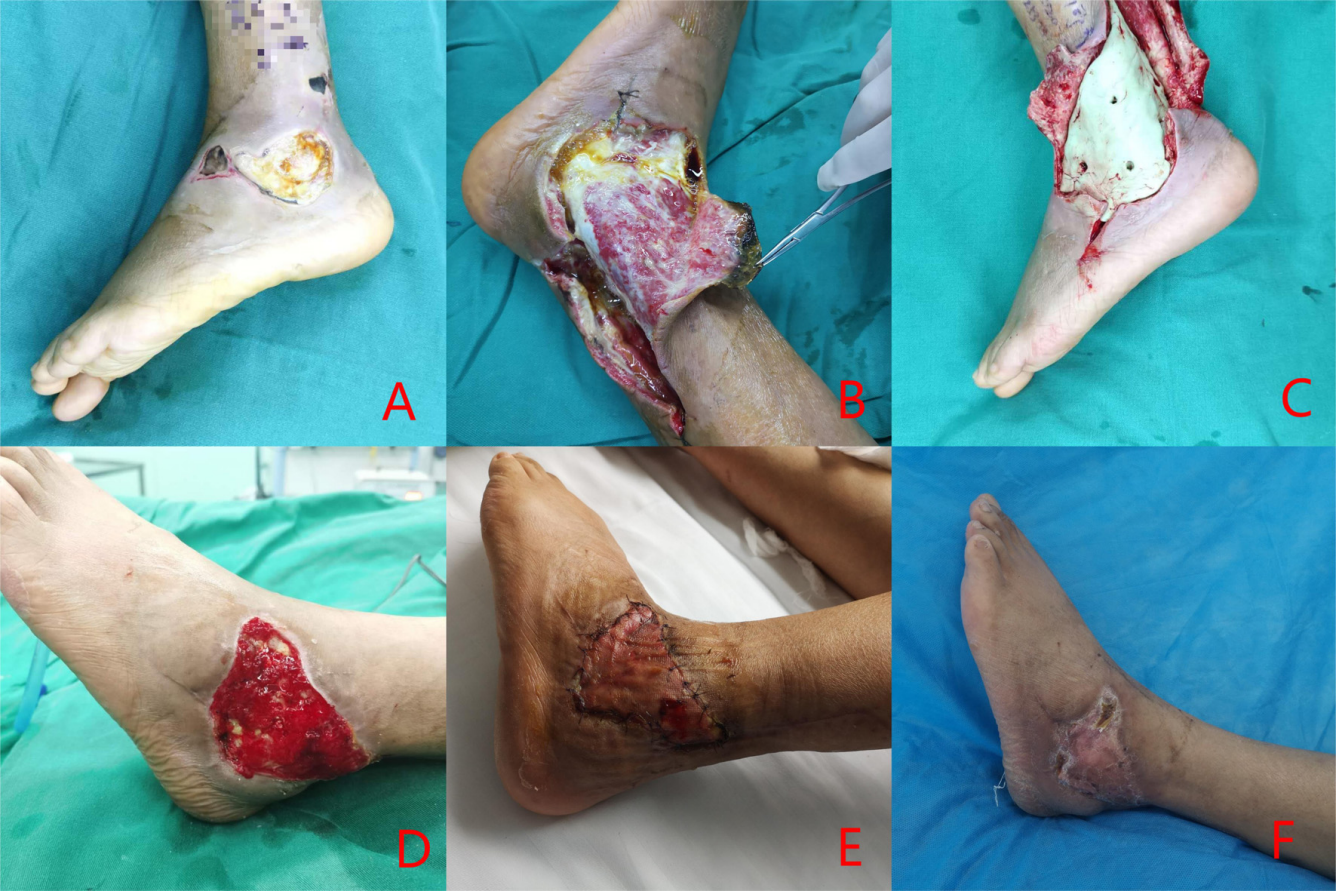
Clinical case: a 54-year-old male diagnosed with Wagner grade 4. **(A)** initial wound prior to debridement; **(B)** debridement procedure, entailing the excision of infected necrotic tissue; **(C)** reconstruction of the defect utilizing antibiotic-impregnated cement; **(D)** removal of the antibiotic cement after three weeks; **(E)** coverage of the wound with an autologous skin graft; **(F)** at follow-up, complete healing of the wound was documented.

**Figure 2:**
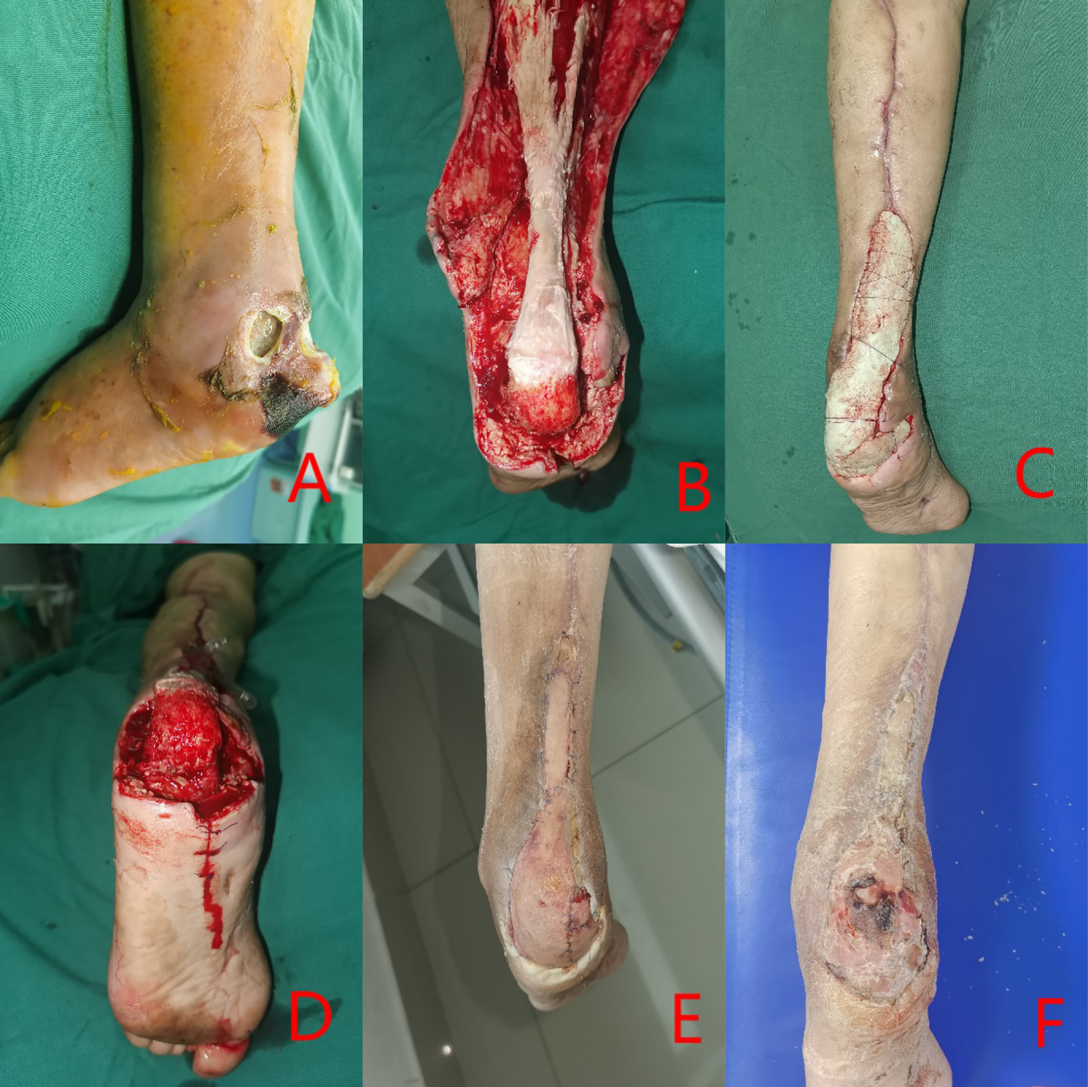
Clinical case: a 73-year-old female diagnosed with Wagner grade 4. **(A)** initial wound prior to debridement; **(B)** Multiple debridement, antibiotic bone cement treatment, entailing the excision of infected necrotic tissue; **(C)** reconstruction of the defect utilizing antibiotic-impregnated cement; **(D)** removal of the antibiotic cement after three weeks; **(E)** coverage of the wound with an autologous skin graft; **(F)** at follow-up, complete healing of the wound was documented.

In this retrospective study, a total of 80 patients with DFUs who met the inclusion criteria were enrolled, consisting of 40 individuals in the experimental group and 40 in the control group. Patients in the experimental group exhibited complete wound healing (**Figures 1**, **2**). The experimental group comprised 22 males and 18 females; their ages ranged from 39 to 86 years, with a mean age of 61.25 ± 11.46 years; the duration of foot ulcers varied from 9 to 34 days, with an average duration of 21.15 ± 4.80 days. The control group included 24 males and 16 females; their ages ranged from 37 to 81 years, with a mean age of 62.34 ± 12.77 years; the duration of foot ulcers spanned from 14 to 31 days, averaging at 19.98 ± 6.91 months. No statistically significant differences were found between the two groups concerning age, gender, HbA1c levels, ABI values, diabetes duration history, or ulcer duration (P>0.05); However, the experimental group exhibited a significantly shorter duration of hospital stay and wound healing time compared to the control group, with a statistically significant difference noted between the two groups (P < 0.05). As presented in **Table 1**.

**Table 1:** Comparison of general condition between two groups of patients.

	Control group (n=40)	Experimental group(n=40)	Statistics	P value
Age	62.34 ± 12.77	61.25 ± 11.46	T=0.435	0.666
Gender (male/female)	24/16	22/18	χ^2=^0. 205	0. 651
Diabetes mellitus history(year)	12.78 ± 6.71	12.55 ± 6.53	T=0.157	0.876
Ulcer area(cm^2^)	8.08 ± 3.74	8.33 ± 3.38	T=0.272	0.787
HbA1c(%)	8.62 ± 1.88	8.57 ± 1.59	T=0.148	0.883
ABI	0.75 ± 0.25	0.76 ± 0.24	T=0.111	0.912
Hospitalization time(day)	30.13 ± 6.16	25.50 ± 6.66	T=3.019	0.004
Wound healing time(day)	34.33 ± 5.96	26.80 ± 5.19	T=5.357	0.000
Cholesterol	4.63 ± 1.18	4.27 ± 1.39	T=1.176	0.247
Triglyceride	1.41 ± 0.50	1.27 ± 0.56	0.245	1.179
Systolic pressure(mmHg)	146.70 ± 21.00	139.28 ± 19.08	1.394	1.171
Diastolic pressure(mmHg)	82.80 ± 8.66	81.85 ± 14.02	0.335	0.739
Ulcer course(day)	19.98 ± 6.91	21.15 ± 4.80	T=0.829	0.412
Wagner classification				
Grade 2	11	8	Z=0.844	0.399
Grade 3	22	23		
Grade 4	7	9		

### 4.2 Results of wound bacterial cultures

The bacterial culture analyses from both groups predominantly revealed Staphylococcus aureus, E.coli, and Pseudomonas aeruginosa as the primary isolates. In the experimental group, there were 16 cases of Staphylococcus aureus, 8 cases of E.coli, and 8 cases of Pseudomonas aeruginosa; additionally, 3 cases of Klebsiella pneumoniae pneumonia subspecies were identified alongside 2 cases each of Staphylococcus epidermidis and Proteus mirabilis,1 cases each of Citrobacter; the control group exhibited 13 cases of Staphylococcus aureus, 11 cases of Escherichia coli, and 5cases each for Pseudomonas aeruginosa and Klebsiella pneumoniae subsp. pneumoniae; furthermore, it included two occurrences each for Staphylococcus epidermidis and Acinetobacter baumannii along with one case each for S. constella subsp constellatus and Proteus mirabilis. Prior to treatment, no significant difference was detected in the number of positive bacterial cultures between the two groups (P>0.05). However, following treatment at days 3, 7, and 15 post-intervention, a significant reduction in positive bacterial cultures was observed for both groups. Notably, the observation group consistently exhibited lower counts than the control group at each time point with statistically significant differences (P<0.05), as presented in **Tables 2** and **3**.

**Table 2:** Comparison of general condition between two groups of patients.

	Control(n=40)	Experimental (n=40)
Staphylococcus aureus	13	16
E. coli	11	8
Pseudomonas aeruginosa	5	8
Klebsiella pneumoniae pneumonia subspecies	5	3
Others	6	5

**Table 3:** Comparison of the number of MDROs cultured in the wound after treatment in the two groups.

	Pre-treatment	Postoperative Day 3	Postoperative Day 7	Postoperative Day 14
Control(n=40)	40	33	24	6
Experimental (n=40)	40	22	12	1
χ^2^		7.040	7.273	6.135
P		0.008	0.007	0.013

### 4.3 A comparison of key indicators before and after treatment in two patient groups

Prior to treatment, no statistically significant differences were observed in inflammatory factors, blood vessel diameter, and VEGF levels between the two groups (P > 0.05). Following treatment, both groups demonstrated improvements in these relevant indicators; notably, the observation group exhibited superior outcomes compared to the control group, with a statistically significant difference noted (P < 0.05), as presented in **Table 4**.

**Table 4:** ** **A comparative analysis of relevant indicators before and after treatment.

	Control group		Experimental group	
	Pre-treatment	Pro-treatment	Pre-treatment	Pro-treatment
ESR(ng/L)	64.75 ± 23.21	19.22 ± 6.79^*^	60.15 ± 30.49	15.28 ± 5.24^*#^
CRP(ng/L)	37.38 ± 14.19	6.58 ± 1.83^*^	36.53 ± 20.70	4.06 ± 1.79^*#^
IL-6(ng/L)	31.03 ± 6.73	14.85 ± 1.93^*^	31.91 ± 7.02	12.34 ± 3.02^*#^
VEGF (ng/L)	85.40 ± 7.52	93.2 ± 5.18^*^	84.93 ± 5.11	96.25 ± 6.4^*#^
Blood vessel diameter(mm)	1.47 ± 0.23	1.82 ± 0.19^*^	1.48 ± 0.28	1.94 ± 0.15^*#^

*P<0.05 compared with the treatment before in this group; #P<0.05 compared with the control group after treatment.

## 5 Discussion

In this retrospective study analysis, we found that ALBC combined with VSD as a treatment for MDROs-DFUs was effective in reducing patient hospitalization time, wound healing time, and greatly reducing the time to conversion of multidrug-resistant bacteria.

In the management of DFUs, most clinicians currently favor surgical resection of infected tissue or bone, complemented by systemic antibiotic therapy during the perioperative period (Ramachandran et al., 2023). For patients with Wagner grade 2 and above, antibiotics alone are insufficient to halt the progression of DFUs; thus, surgical debridement emerges as the most effective intervention. However, in cases involving wounds infected with multi-drug resistant bacteria, mere debridement may not facilitate prompt wound healing and could potentially exacerbate drug-resistant bacterial transformation. Despite adherence to established principles for DFUs management, there remains significant potential for enhancing treatment outcomes.

The management of MDROs-DFUs presents a significant challenge in clinical practice. The conventional treatment approach involves staged debridement and dressing changes until the wound granulation is sufficiently healthy, followed by skin grafting or flap reconstruction for wound repair. During the treatment process, if the integrity of the skin and soft tissue cannot be restored promptly, leading to the formation of multi-drug resistant wounds, it becomes crucial to effectively control infection and facilitate timely wound healing. However, traditional methods exhibit several limitations including prolonged duration of treatment, challenges in infection control, numerous complications, and suboptimal functional outcomes in later stages (Barbier and Timsit, 2020).

The advancements in materials engineering and tissue engineering have significantly broadened clinical perspectives and methodologies. The implementation of localized sustained-release systems, which utilize specialized materials containing load-sensitive antibiotics, has emerged as an optimal solution for challenging wounds affected by MDROs bacteria. In cases of severe soft tissue infections, the combination of ALBC and VSD is frequently considered the preferred treatment strategy (Yang et al., 2023). Over the past decade, retrospective analyses and Meta-Analysis, it can be concluded that topical antibiotic bone cement treatment significantly shortened the wound healing time and reduced the number of debridements in patients with DFUs without increasing the rate of complications, and that topical antibiotic bone cement has become a commonly used surgical option for the treatment of DFUs (Chen et al., 2024; Ding et al., 2022; Dong et al., 2023; Ramachandran et al., 2023; Tarabichi and Parvizi, 2023; Wu et al., 2024; Zhong et al., 2024). However, due to local microcirculation disorders and the blood-bone barrier present in DFU patients, antibiotic penetration at the infection site is suboptimal (Lew and Waldvogel, 2004), making it challenging to achieve the necessary Minimum Inhibitory Concentration (MIC) in both soft and bone tissues. Additionally, prolonged treatment durations and other contributing factors complicate the assessment of clinical efficacy for these antibiotics (Nandi et al., 2016). Insufficient vascular perfusion further hinders antibiotic penetration, diminishing their antibacterial effectiveness even when administered at standard dosages (Hart et al., 2017). As a localized sustained-release system for antibiotics, antibiotic-loaded bone cement offers advantages such as precise targeting, elevated local drug concentrations, and reduced resistance, thereby enhancing the control of wound infections. In recent years, this formulation has been extensively utilized by researchers both domestically and internationally to address various wound infections and refractory osteomyelitis (Mendame et al., 2021; Wang et al., 2023). Furthermore, it was observed that the duration of negative bacterial cultures, the time required for wound healing, and the length of hospital stays in patients receiving vancomycin-loaded bone cement were significantly superior compared to those in the control group. ALBC has various distinguishing features: (1) The formation of a biological membrane improves wound healing: Masquelet et al. (Masquelet et al., 2000) were the first to discover that applying bone cement to a wound causes the creation of a biological membrane known as the Induced Membrane (IM). This membrane promotes wound healing by releasing TGF-β1 and VEGF (Giotikas et al., 2019). Histopathological investigations indicate that IM has biological activity, with released substances aiding wound healing, increasing angiogenesis, and maybe contributing to bone formation (Chopra et al., 2023; Fischer et al., 2016). DFUs are frequently associated with pathological diseases such as blockage of tiny arteries and capillaries in the lower limbs (Quilici et al., 2016). (2)The utilization of localized high-concentration antibiotics is associated with a diminished occurrence of adverse effects: The vascular impairment observed in the feet of patients with DFUs results in diminished peripheral perfusion, consequently leading to reduced concentrations of antibiotics within both soft and bone tissues, thereby limiting their therapeutic efficacy. Furthermore, the development of foot ulcers contributes to a decrease in fresh and viable granulation tissue surrounding the wound, which hinders effective proliferation and ultimately results in suboptimal wound healing. ALBC not only effectively eradicates bacteria within vascularized tissues but also exhibits bactericidal activity against surface bacteria devoid of a blood supply. Systemic intravenous administration of antibiotics is insufficient for penetrating local lesions, resulting in suboptimal bactericidal concentrations at the infection site. The utilization of local antibiotic bone cement facilitates continuous and sustained release of antibiotics, directly targeting the lesion area to achieve bacterial eradication, thereby improving the infection cure rate (Leta et al., 2024; Mendame et al., 2021). Furthermore, the occlusive effect of antibiotic cement effectively eliminates dead spaces within the infected lesion, thereby preventing pus accumulation and further inhibiting bacterial proliferation, which markedly reduces the turnaround time for bacterial culture. This benefit remains unparalleled by systemic antibiotics (Dong et al., 2023). Ideally, the most suitable local antibiotic should be selected based on bacterial culture results from the target site; however, in clinical practice, due to the imperative for prompt surgical intervention in DFUs to enable debridement and control infection, preoperative sensitivity testing is frequently impractical. Consequently, vancomycin is generally considered as the first-line therapeutic option (Guo et al., 2023; Wu et al., 2024).

Vancomycin has a broad spectrum of susceptibility and kills common pathogenic microorganisms (Alvarez et al., 2016). Both domestic and international studies have shown that in bacterial cultures derived from patients with DFUs, Staphylococcus and Enterobacter are predominant, exhibiting no resistance to vancomycin, which is consistent with previous research findings (Ghosh et al., 2022; Guo et al., 2023). Multiple investigations indicate that vancomycin ranks among the most effective antibiotics for managing infections in DFUs patients, particularly against Staphylococcus species and other Gram-positive bacteria (Davani et al., 2021; Wu et al., 2018). Its unique physicochemical properties ensure optimal diffusivity and stability of the antibiotic within local tissues, which is critical for its localized application. Moreover, vancomycin is commonly employed as an additive in antibiotic-loaded bone cement, releasing local concentrations of approximately 0.5-2.0 g/mg to meet MIC requirements (Patel et al., 2009). Through the micropores of the bone cement, vancomycin is gradually released into surrounding tissues, resulting in tissue concentrations significantly higher than those achieved through intravenous or oral administration; this enhances control over local ulcer wound infections.

The Mechanism of Action and Advantages of VSD: (1) Reduction of Inflammation and Promotion of Granulation Tissue Growth: Chen et al. (Chen et al., 2019) demonstrated that negative pressure drainage (VSD) facilitates the migration, division, and proliferation of tissue cells within the wound environment, activates intracellular signaling cascades, stimulates endothelial cell proliferation, and promotes angiogenesis in the affected area. This process enhances blood circulation in the wound site and accelerates self-repair. (2) Improvement of Microcirculation and Local Immune Status: VSD enhances revascularization by increasing capillary diameter and blood volume to improve local capillary density, stimulate endothelial cell proliferation, and promote neovascularization. Consequently, this process is characterized by a reduction in vascular resistance, an increase in flow velocity, restoration of microvascular basement membrane integrity, decreased intercellular spacing, and reduced vascular permeability—thereby alleviating tissue edema (Li et al., 2017). (3) Elimination of Bacteria from Wounds: In chronic bacterial infection healing processes, wounds are often compromised by pathogenic bacteria. The colonization by these bacterial populations results in prolonged stagnation at the inflammatory stage without progression into proliferation or repair phases. In contrast, VSD significantly reduces bacterial proliferation and dissemination within wounds while effectively inhibiting biofilm formation (Kumar et al., 2018). (4) Inhibition of Apoptosis and Acceleration of Nerve Repair: VSD also exerts a significant effect on relieving muscle spasms as well as promoting nerve damage repair. Younan et al. (Younan et al., 2010) suggest that this technique regulates the regeneration process for damaged nerve fibers by inducing expression levels of neurotrophic factors and neuropeptides—effectively modulating recovery for injured nerve fibers.

The Advantages of Combining Vancomycin Bone Cement with VSD: (1) The VSD technology provides sustained negative pressure, enhances blood circulation, and accelerates the wound healing process. Simultaneously, vancomycin bone cement demonstrates a significant antibacterial effect that aids in reducing bacterial load and decreasing the incidence of infections, thereby facilitating skin healing—particularly in DFUs and other susceptible wounds (Chen et al., 2023; Yang et al., 2024). (2) The combined application of VSD and vancomycin bone cement is suitable for various stages of diabetic foot ulcers, including Wagner grades II to IV. This synergistic approach can expedite wound healing while enhancing treatment efficiency, reducing the frequency of VSD replacements, and consequently shortening patients’ hospital stays. Moreover, this combination therapy may also mitigate the risk of complications such as infection spread and ulcer deterioration (Sun et al., 2022). (3)This combined treatment improves patients’ quality of life by minimizing infections and accelerating wound healing. Although both vancomycin bone cement and VSD incur higher costs, the reduction in hospital stay duration coupled with enhanced treatment efficacy may yield long-term economic benefits.

## 6 Conclusion

In summary, the combined treatment of ALBC and NPWT for MDROs-DFUs not only significantly shortens the hospital stay and the time for negative MDROs, but also reduces the patient’s pain and burden. Furthermore, this method helps promote postoperative body recovery, improve local blood supply, effectively reduce inflammatory reactions, and accelerate wound healing.

## 7 Limitations

This study is a retrospective analysis, and there are certain limitations. At the same time, the sample size in this study is small, and more random prospective controlled studies are needed to further verify these issues. Currently, there are no multi-center randomized controlled trial results. In addition, due to the lack of long-term follow-up data, the long-term follow-up results may be different. These are the areas that need to be further improved in future studies to better serve clinical practice.

## Data Availability

The original contributions presented in the study are included in the article/supplementary material. Further inquiries can be directed to the corresponding authors.
